# Quantification of 20-Hydroxyecdysone From *Drosophila* Larvae at the Onset of Metamorphosis

**DOI:** 10.21769/BioProtoc.5831

**Published:** 2026-10-05

**Authors:** Jyotsna Kawadkar, Lisupriya Baral, Ram Kumar Mishra

**Affiliations:** Department of Biological Sciences, Indian Institute of Science Education and Research, Bhopal, India

**Keywords:** 20-Hydroxyecdysone, Metamorphosis, *Drosophila*, Horseradish Peroxidase, ELISA

## Abstract

Metamorphosis in class Insecta is defined as the drastic transition of an organism from a larval stage to a morphologically very different adult form. Insects such as *Drosophila melanogaster* undergo metamorphosis, which requires precise temporal control, exerted by ecdysteroids, the primary regulators of these developmental transitions. The active form of ecdysone is 20-hydroxyecdysone (20E), which acts in pulses that trigger the onset of different developmental stages. A massive pulse of 20E occurs between the prepupal and pupal stages, triggering pupariation. Precise quantification of 20E levels is critical for understanding how this hormonal signaling governs the developmental timing of different stages. This protocol is sensitive for detecting low levels of 20E and describes a methanol-based extraction method coupled with a competitive ELISA to quantify whole-body 20E levels in third-instar *Drosophila* larvae. Further, this protocol enables detection of differences in ecdysone titers among genotypes affecting ecdysone biosynthesis, particularly at the late third-instar larval stage. This protocol can also be utilized for disease-causing insects (*Aedes aegypti*), as well as economically relevant beneficial insects (*Apis mellifera*), to study the effects of ecdysteroid analogs during development.

Key features

• 20E-dependent Ecdysone signaling induces morphological changes in insects known as metamorphosis.

• This protocol provides details for 20E quantification, based on the competitive ELISA between unconjugated and HRP-conjugated 20E.

• Spectrophotometry-based color intensity is directly proportional to the conjugated 20E and inversely proportional to the amount of 20E present in the sample.

## Graphical overview


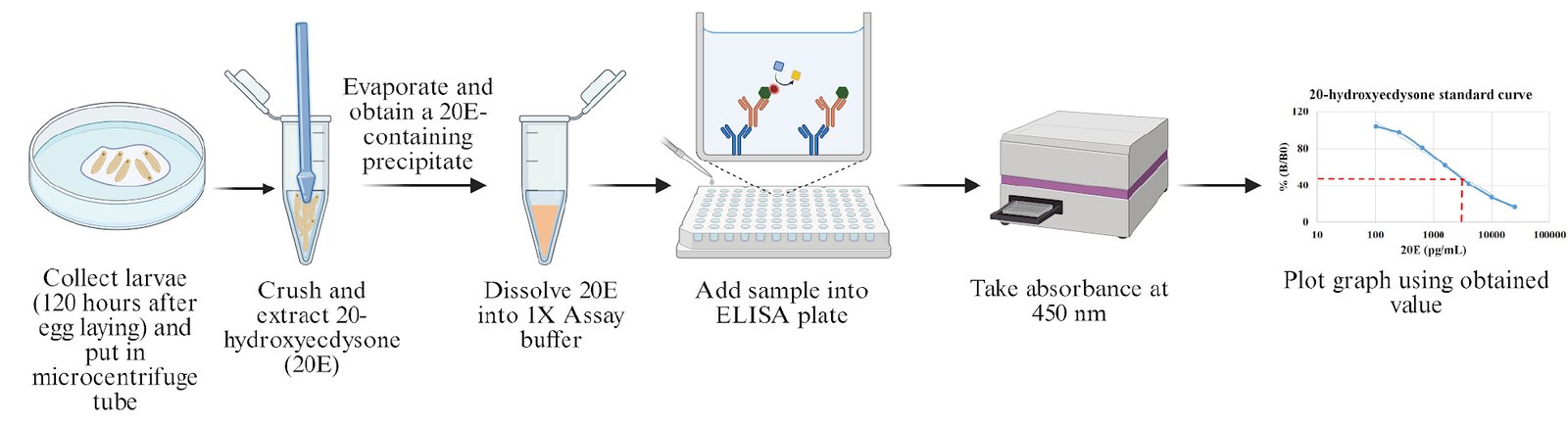


**Schematic flowchart for 20E estimation**

## Background

Organisms belonging to the class Insecta utilize steroid hormones across various developmental stages to regulate their growth and maturation. These steroid hormones govern key life-stage transitions, such as the crucial larval-to-pupal metamorphosis. *Drosophila* is a powerful model organism for studying developmental changes. In *Drosophila*, metamorphosis is initiated by precisely timed pulses of the steroid hormone ecdysone, produced from the prothoracic gland (PG) attached to the larval brain through a series of enzymatic processes [1,2]. The biosynthesis of ecdysone in the PG is primarily regulated by the neuropeptide prothoracicotropic hormone (PTTH). The binding of PTTH signals through the receptor tyrosine kinase Torso, activating the Ras-MAP kinase pathway and driving the transcription of the *Halloween* gene and ecdysone production. The enzymes involved in ecdysone synthesis are encoded by the *Halloween* genes (*spookier, phantom, disembodied*, and *shadow*) [3–5]. Disruption of this signaling axis at any level results in reduced ecdysone levels and consequent developmental delay or arrest [6,7].

Ecdysone is subsequently secreted into the hemolymph from the PG and converted to its active form, 20-hydroxyecdysone (20E), through the action of the *shade* gene product in the peripheral tissues. In target tissues, 20E binds to the ecdysone receptor (EcR), which translocates into the nucleus and activates downstream target genes, which initiate the transcriptional cascade required for the larval-to-pupal transition [8].

We have shown that Nup107, a nuclear pore complex member, acts as an upstream epistatic regulator of Torso-mediated ecdysone signaling in *Drosophila* [6]. RNAi-mediated depletion of Nup107, either ubiquitously (using *Actin5C-Gal4*) or specifically in the prothoracic gland (using *Phm.Gal4*), led to a striking reduction in whole-body 20E levels, particularly at 120 h after egg laying (120 h AEL), which coincides with the critical pre-pupariation 20E pulse [6].

To accurately measure the 20E titer across genotypes, a robust and sensitive quantification method is essential. Although several methods, such as LC-MS/MS, GC-MS, and radioimmunoassay, are available for 20E detection, they often present limitations; radioimmunoassay involves hazardous radioactive components, while other techniques may require complex instrumentation and may not be quantitative. Competitive ELISA circumvents all the shortcomings of available methods. The most effective one relies on competitive ELISA, where a competition between the endogenous 20E present in the sample and a horseradish peroxidase (HRP)-conjugated 20E antigen (20E-HRP) for binding to a fixed amount of anti-20E antibodies is quantified. As a result, if the sample has a high amount of competing 20E, less 20E-HRP binds to the antibodies. Due to this, the signal generated is inversely proportional to the 20E concentration in the sample. This allows quantification against a standard curve ranging from 100 to 25,000 pg/mL. The kit demonstrates high specificity for 20E due to a highly specific antibody against 20E, with minimal cross-reactivity to precursor steroid, ecdysone, and related steroids such as Makisterone A or Ponasterone A. These attributes make it well-suited for *Drosophila* developmental studies, distinguishing 20E from structurally related ecdysteroids.

This protocol outlines the collection of high-quality samples from developmentally synchronized larvae, followed by the extraction of 20E from whole larvae. We detail the principles of competitive enzyme-linked immunosorbent assay (ELISA) for ecdysone quantification using a commercially available enzyme immunoassay kit. This particular kit utilizes an HRP-mediated enzymatic cascade with readily accessible substrates such as tetramethylbenzidine (TMB).

## Materials and reagents

**Biological materials**

This protocol uses *Drosophila melanogaster* as the biological material.

1. *w^1118^ (BDSC:3605; FLYB: FBst0003605; RRID:BDSC_3605/*origin: Bloomington Drosophila Stock Center)

2. *Nup107^KK^ RNAi, P{KK108047}VIE-260B (VDRC: v110759; FLYB: FBst0482324; RRID:FlyBase_FBst0482324/*origin: Vienna Drosophila Resource Center)

3. Phm.Gal4, y[1 ] w[*]; P{w[+mC]=phtm-GAL4.O}22 (BDSC:80577; FLYB: FBti0201787; RRID:BDSC_80577/origin: Bloomington Drosophila Stock Center)

**Reagents**

1. Corn flour (Gangwal Makka Aata Yellow Maize Flour, Gangwal Flour Foods LLP)

2. Table sugar (Madhur-Pure & hygienic Sugar, Amazon)

3. Yeast extract (HiMedia, catalog number: RM027)

4. Agar (Merck, catalog number: A5306)

5. Dextrose (HiMedia, catalog number: GRM077)

6. Methyl-4-Hydroxybenzoate (Sigma-Aldrich, catalog number: H5501)

7. Ortho-phosphoric acid (Merck, catalog number: 100573)

8. Propionic acid (HiMedia, catalog number: GRM3658)

9. Methanol (Merk, catalog number: 1.94516.2521)

10. Liquid nitrogen (IISER Bhopal, in-house facility)

11. 20-Hydroxyecdysone ELISA kit (Thermo Fisher Scientific, catalog number: EIAHYD); keep at -20 °C until opened; once opened, store at 4 °C and use within 2 weeks

12. Assay buffer concentrate (5×) (Thermo Fisher Scientific, catalog number: EIAHYD)

13. Wash buffer concentrate (20×) (Thermo Fisher Scientific, catalog number: EIAHYD)

14. 20-Hydroxyecdysone standard; 2,500 ng/mL (Thermo Fisher Scientific, catalog number: EIAHYD)

15. 20-Hydroxyecdysone antibody (Thermo Fisher Scientific, catalog number: EIAHYD)

16. 20-Hydroxyecdysone conjugate (Thermo Fisher Scientific, catalog number: EIAHYD)

17. Tetramethylbenzidine (TMB) substrate (Thermo Fisher Scientific, catalog number: EIAHYD)

18. Stop solution; contains 1 M HCl (Thermo Fisher Scientific, catalog number: EIAHYD)

19. Distilled water

**Solutions**

1. Assay buffer (1×) (see recipes)

2. Wash buffer (1×) (see recipes)

3. Fly food ingredients (see recipes)

**Recipes**

**1. Assay buffer (1×)**

**Table:** 

Reagent	Final concentration	Volume
Assay buffer concentrate (5×)	1×	10 mL
Distilled water	-	40 mL
Total		50 mL

*Note: Always prepare the assay buffer solution fresh, immediately before use. Keep at 4 °C and use within 2 weeks of opening.*

**2. Wash buffer (1×)**

**Table:** 

Reagent	Final concentration	Volume
Wash buffer concentrate (20×)	1×	5 mL
Distilled water	-	95 mL
Total	-	100 mL

*Note: Always prepare the wash buffer solution fresh, immediately before use.*

**3. Fly food ingredients**

**Table:** 

Ingredient	Amount/L
Corn flour	80 g
Sugar	40 g
Dextrose	20 g
Yeast extract	15 g
Agar	10 g
Methyl-4-hydroxybenzoate	1 g
Propionic acid	4 mL
Orthophosphoric acid	0.6 mL
Ethanol absolute	10 mL

*Notes:*

*1. All components (corn flour, sugar, dextrose, yeast extract, and agar) are dissolved in distilled water and autoclaved at 121 °C for 20 min. Let it cool down to 50–55 °C (check it with a thermometer), then add methyl 4-hydroxybenzoate solution, propionic acid, and orthophosphoric acid to the media. Mix it thoroughly and pour 8–10 mL into each glass vial.*

*2. While handling chemicals (methyl-4-hydroxybenzoate, propionic acid, and orthophosphoric acid), always wear appropriate PPE, including a lab coat, safety goggles, and chemical-resistant gloves.*

**Laboratory supplies**

1. 1.5 mL microcentrifuge tubes (Genaxy, catalog number: GEN-MT-150-C-S)

2. Glass vials (test tube heavy flat bottom 24 mm × 90 mm, Shiv Enterprise Bhopal, India)

3. 0.5–10 μL micropipette tips (Axygen Scientific, catalog number: T-300-L-R-S)

4. 1–200 μL micropipette tips (Axygen Scientific, catalog number: TR-222-C-L-R-S)

5. 100–1,000 μL micropipette tips (Axygen Scientific, catalog number: T-1000-C-L-R-S)

6. 35 mm dish (Eppendorf, catalog number: CC7682-3340)

7. 100 mm dish (Eppendorf, catalog number: CC7682-3394)

8. Food bottles (Tarson, catalog number: 441120, CONICAL FLASK 250 mL PP)

9. CO_2_ pads (Tritech Research, model: MINJ-DROS-FP)

10. Cotton plugs (non-absorbent cotton wool) (Mamta Spinners Pvt. Ltd. Gulabpura, Rajasthan, India)

11. *Drosophila* Sorting Brush, pack 12 (Carolina)

12. Plastic pestles for micro-tube homogenizer system (Merk, catalog number: BAF650009002)

13. Kimwipes (Kimtech Science, catalog number: 34120)

14. Parafilm (Merck, catalog number: HS234526B)

15. -80 °C deep freezer (Thermo Scientific)

16. KC Purple Nitrile Gloves-M (Kimberley, catalog number: 55082)

## Equipment

1. Micropipette (Nichipet EXII, and Corning)

2. BOD Incubators (Meta Lab Scientific Industries, model: MSI-9)

3. Millipore water purification unit (Merck, Progard TS2, catalog number: PR0G0T0S2)

4. Leica Ivesta 3 (Leica microsystems, model: Ivesta 3)

5. Stereomicroscope (Leica, model: S6E)

6. Tabletop centrifuge (Eppendorf, model: 5424, Thermo Scientific MicroCL 21R)

7. Vacuum concentrator plus with Rotor F-45-48-11 (Eppendorf, catalog number: 5305000568)

8. Plate reader (BioTek, model: EON)

9. Ultra micro balance (Mettler Toledo, catalog number: ME204)

10. ELISA plate shaker (Neuation, model: iSHAK TS4 NXT)

## Software and datasets

1. BioTek Gen5 software (version 2.01.14) (used with ELISA plate reader)

2. Microsoft Excel Professional Plus 2013 (used for standard curve fitting)

## Procedure

**A. Genetic crosses**

1. From the parent vial/bottle, collect virgin females with appropriate Gal4 (*Phm.Gal4*) into a fresh vial.

*Note: Vials with virgins can be maintained at 18 °C if required.*

2. Collect males of the appropriate genotypes, *w^1118^* and *UAS-Nup107^KK^*, to perform the RNAi-mediated knockdown [9], and store them at room temperature.

3. Add 10–12 males of appropriate genotype (*w^1118^* and *UAS-Nup107^KK^*) in vials with 30–35 virgin females of *Phm.Gal4* genotype.

4. Label the vials for the cross type and date and keep the crosses at room temperature for 24 h.

5. Incubate crosses in an appropriate temperature-controlled incubator (29 °C).

6. Perform each cross independently three times for biological triplicates at 29 °C.

**Figure 1.**
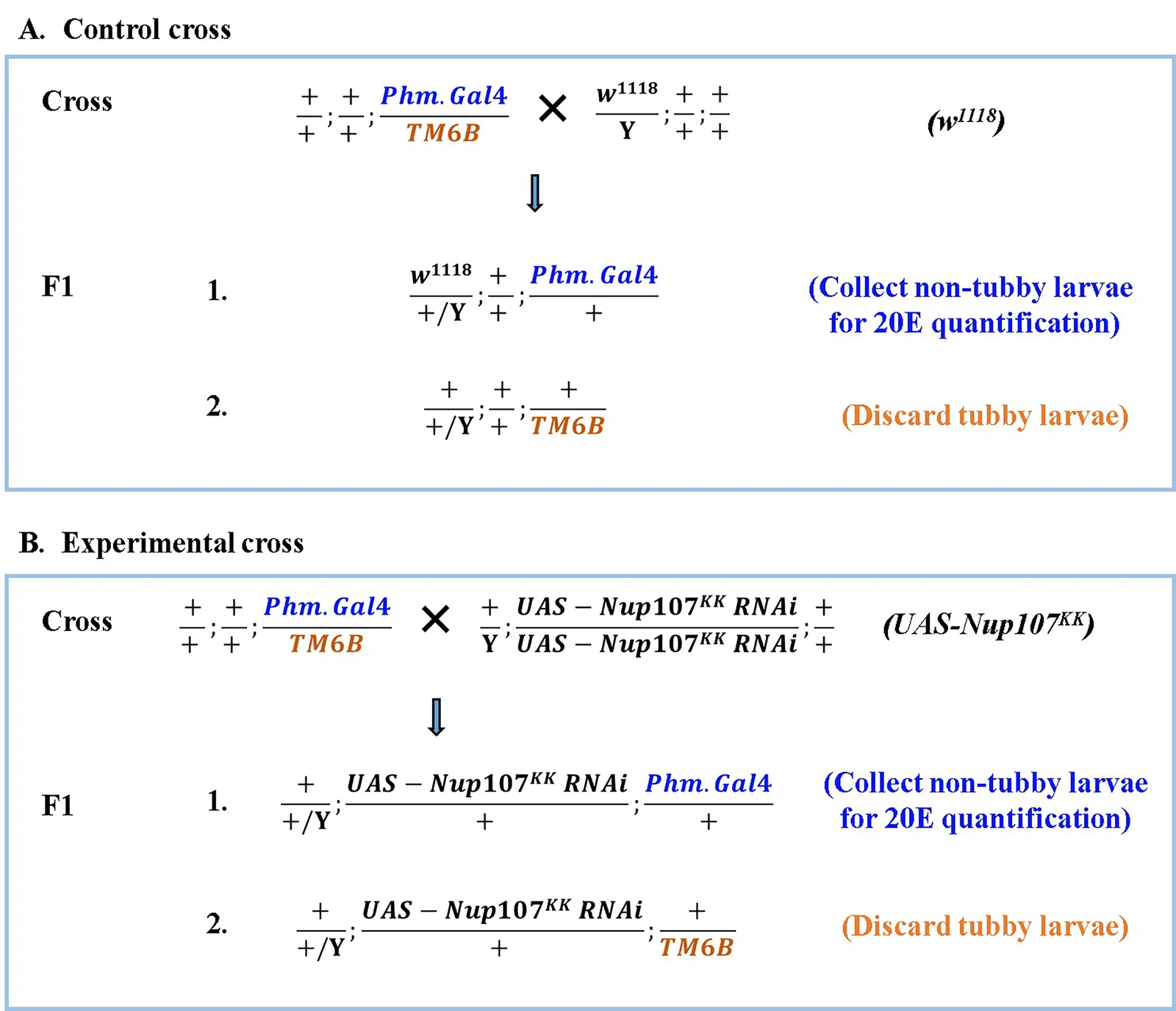
Schematic of a *Drosophila* genetic cross. The control and experimental cross scheme. For comparison, *Phm.Gal4* virgin females crossed with males of *w^1118^* as the control cross (A, upper panel) and *UAS-Nup107^KK^* RNAi as the experimental cross (B, lower panel). Collect non-tubby F1 progeny as the desired phenotype.

**B. Collection and synchronization of larvae**

1. After 24 h of incubation of the cross at 29 °C, transfer flies (crosses shown in Figure 1) into a fresh media vial and keep them for egg laying.

2. Collect flies after 3 h of egg laying and put them again in another fresh vial for the next round of egg laying.

3. Repeat steps B1–2 for more egg laying.

*Note: Ensure to collect 100–150 eggs from each cross.*

4. After that, transfer flies into a new vial for storage/maintenance.

5. For synchronization, label each of the new vials appropriately in a time-specific manner.

*Note: The synchronization process will help to collect the late third-instar larvae (120 h AEL).*

6. When larvae hatch from these egg-laying vials, they are age-matched.

7. Based on the developmental timing illustrated in Figure 2, collect the non-tubby late third-instar larvae (120 h AEL) for 20E measurement (Figure 3).

*Note: Keep the larvae from each 3-h collection vial separate, and do not combine them. Each sample should contain only 16–18 larvae (25 mg) per genotype.*

**Figure 2.**
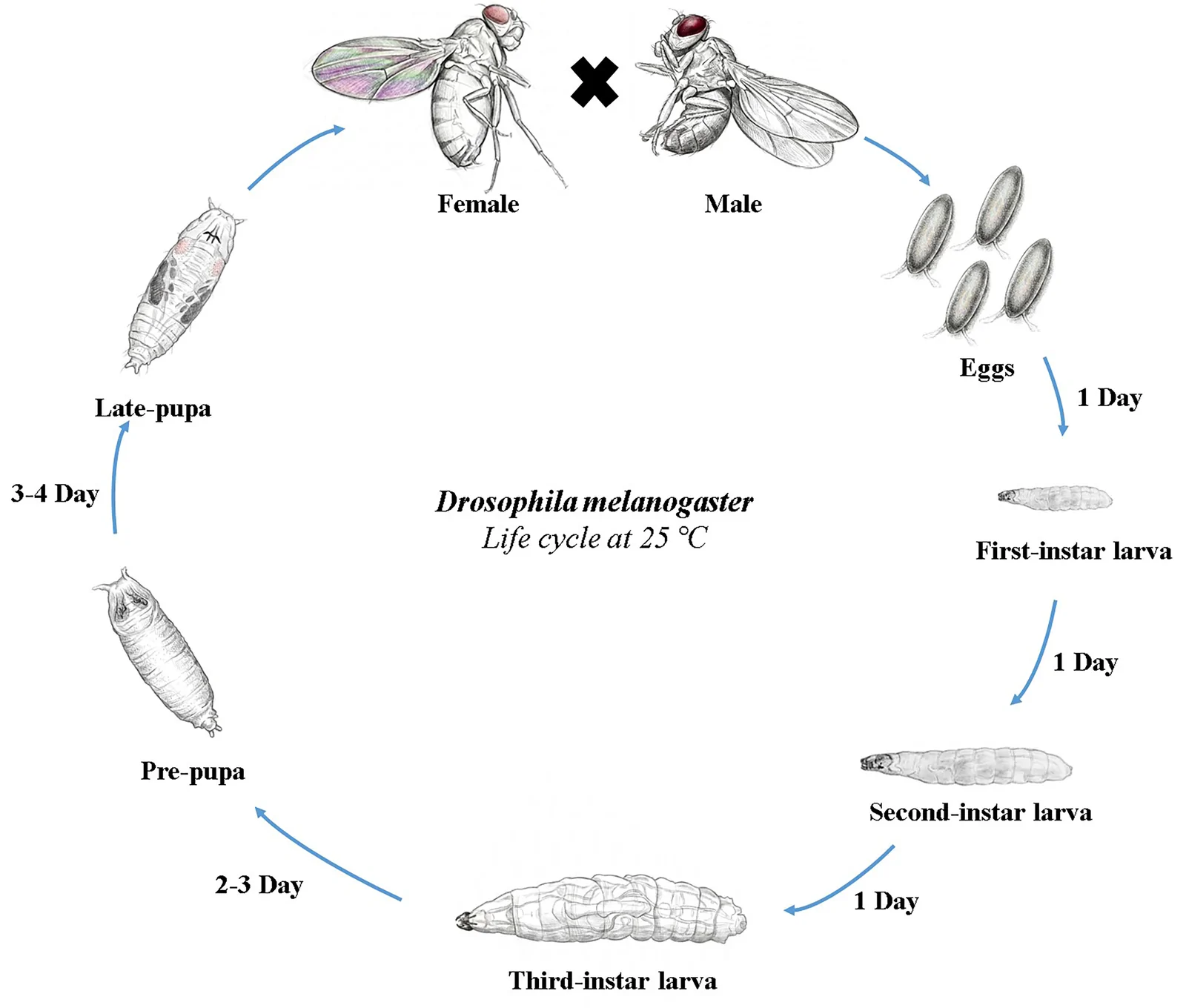
*Drosophila melanogaster* life cycle at 25 °C. The life cycle begins when a male and female adult mate, and the female lays fertilized eggs (embryonic stage). There are three larval stages, and the third-instar larvae feed vigorously for approximately 2–3 days, during which they continue to grow and store energy in preparation for the next stage, the pupal stage. Once the third-instar larvae have fully developed, they move away from their feeding area to find a safe place where they can start the pupation process. The pupal stage lasts approximately 3–4 days, after which the pupae emerge as adult fruit flies.

**Figure 3.**
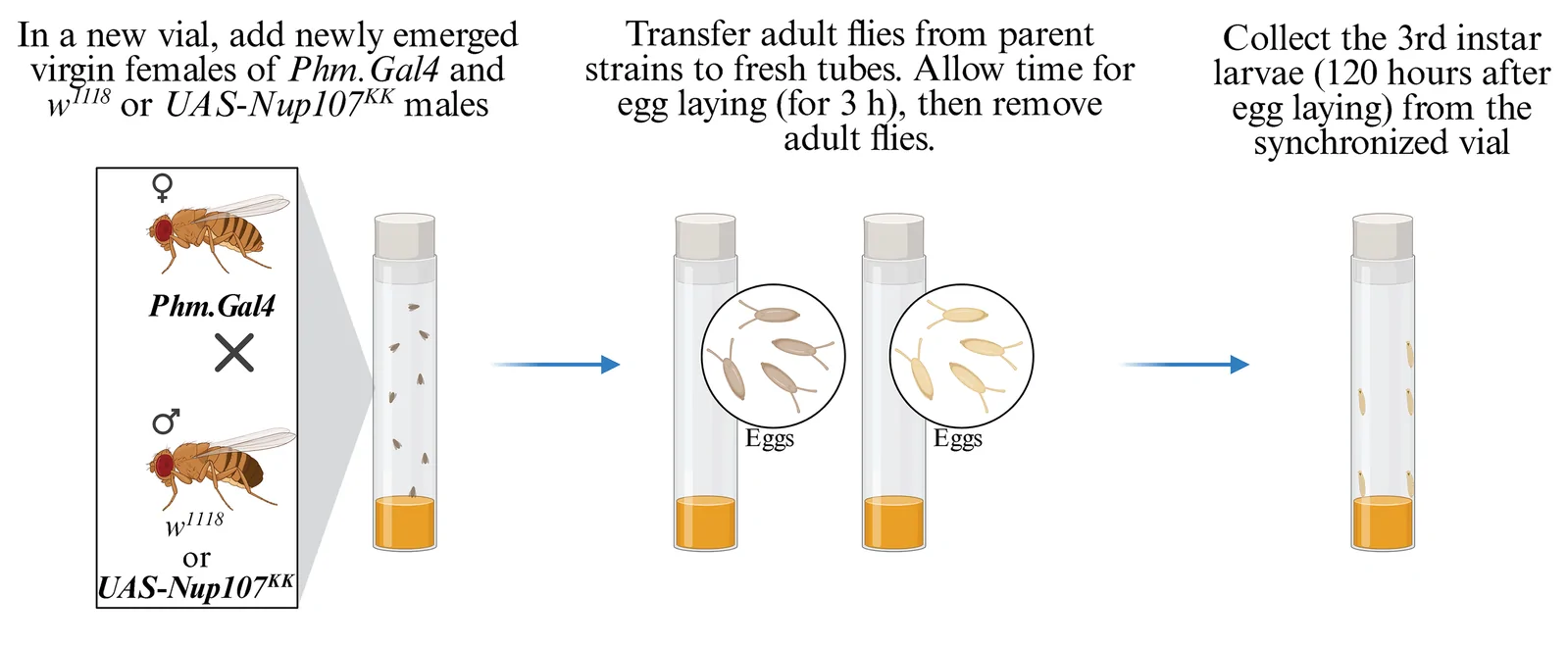
Illustration of synchronization of egg laying for the collection of late third-instar larvae. Crosses are established, and adult flies are transferred to a fresh vial for a 3-h window to synchronize egg deposition, ensuring uniform developmental stages for subsequent larval collection.

**C. Standard sample preparation for ecdysone measurement**

1. As shown in Figure 4, take 10 μL of 20-Hydroxyecdysone (20E) standard (2,500 ng/mL) solution into a 1.5 mL microcentrifuge tube (MCT) with a 10 μL pipette.

2. Add 990 μL of 1× assay buffer to make the 25,000 pg/mL standard solution. Label it properly to avoid any confusion. Mix by vortexing for later use.

3. For the serial dilution process, add 300 μL of 1× assay buffer to seven different 1.5 mL MCT and label them as per Figure 4.

4. The concentrations of 20E in these are as follows: 25,000, 10,000, 4,000, 1,600, 640, 256, 102.4, and 0 pg/mL.

5. Now, add 200 μL of 25,000 pg/mL standard solution (Std1) into the MCT containing 300 μL of 1× assay buffer, and label it as 10,000 pg/mL (Std2).

6. Use the same process to make the successive dilutions of the standard to generate Std3-Std7 as shown in Figure 4.

7. Mix the contents of each tube thoroughly by vortexing between steps.

**Figure 4.**
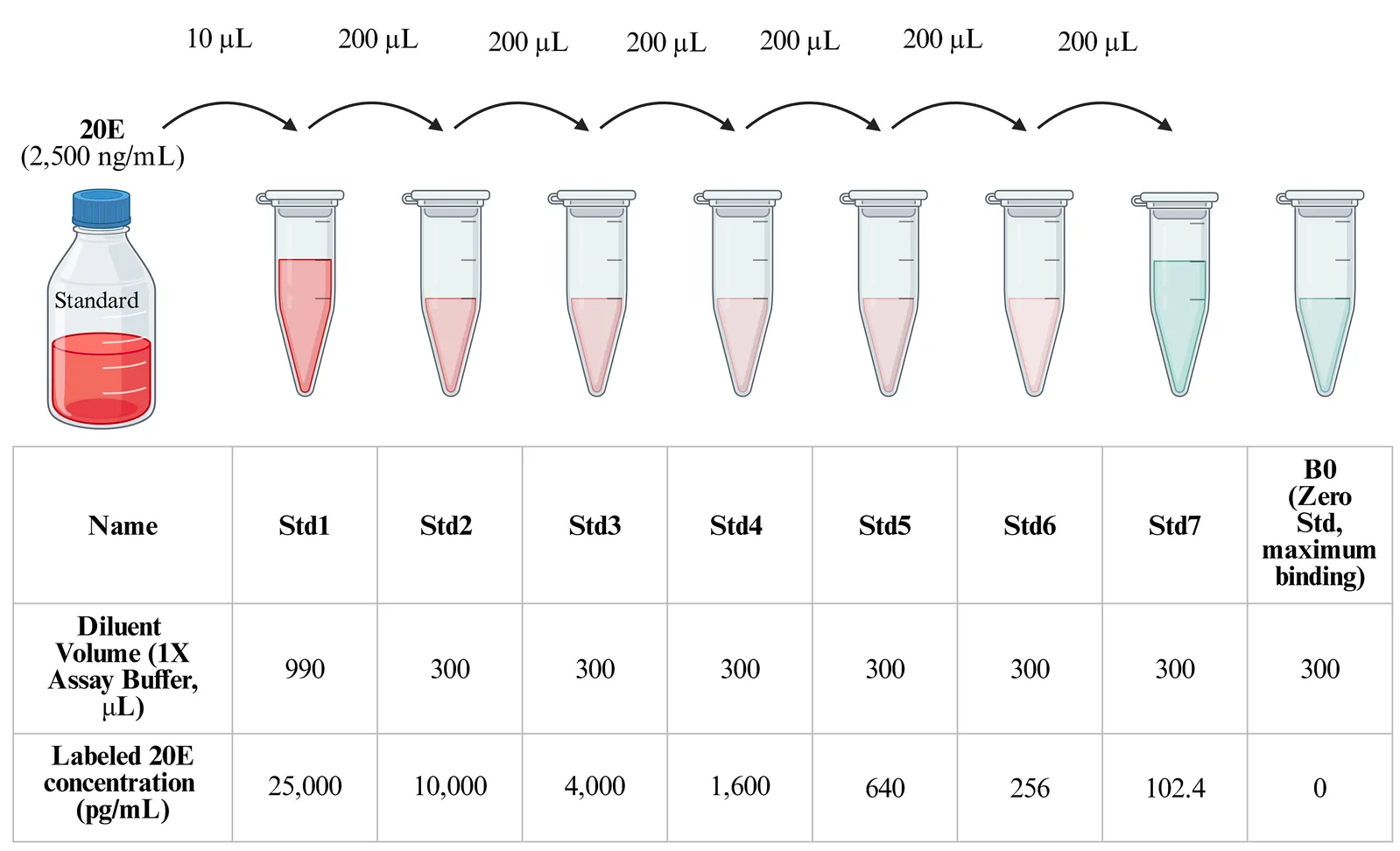
Standard 20-Hydroxyecdysone (20E) dilution for making a standard curve. Preparation of different standard solutions (Std1–7 and B0) of 20E for ELISA using 1× assay buffer as diluent.

**D. Sample preparation for ecdysone measurement**

1. Using a paintbrush, transfer the larvae (120 h AEL) into a bubble of distilled water on a plate. To wash them twice, move the larvae into a new distilled water bubble to remove food and debris from the cuticle surface.

2. Give a final wash or rinse with distilled water, then dry the larvae with a lint-free wipe (Kimwipe) by keeping them on the wipe with the help of a paintbrush for 1 min.

3. Take a 1.5 mL MCT and put it on the ultra-micro balance, then reset to zero by pressing the Tare button. Now, put the larvae (washed and dried in the previous step) in MCT and weigh 25 mg of larvae (16–18 larvae; note down the exact weight) for each biological replicate.

4. Repeat this weighing process for each genotype at 120 h after egg laying (AEL).

*Note: Use a paintbrush rather than forceps for washing, transferring larvae between locations, and moving them into MCTs. This prevents accidental damage to the larvae.*

5. Flash-freeze the weighed and dried larvae immediately in liquid nitrogen and store at -80 °C until further use.

6. For 20E extraction, take the larvae-containing MCT and crush the larvae in 300 μL of ice-cold methanol with a homogenizer (a plastic pestle).

*Note: For frozen samples (-80 °C), thaw the larvae slightly on ice before the addition of methanol. Methanol is toxic and flammable; handle strictly inside a chemical fume hood using gloves and eye protection, away from open flames.*

7. Centrifuge the sample at 17,000× *g* for 10 min at 4 °C and collect the supernatant.

8. Divide the supernatant by transferring 150 μL into each of two separate MCTs to facilitate faster and more efficient evaporation. The supernatant will contain the extracted 20E.

9. Seal the top of the MCT with parafilm and make perforations for efficient evaporation (see Figure 5).

10. Evaporate methanol from both tubes by vacuum centrifugation for 60 min. Increase the centrifugation time if residual solution remains.

*Note: Ensure methanol is completely evaporated; any residual methanol may interfere with the accurate measurement of 20E concentration.*

11. Repeat the centrifugation until all liquid has evaporated. After evaporation, store the sample at -80 °C for further use.

12. Dissolve the pellet by adding 200 μL of 1× assay buffer to one of the two pellet-containing tubes.

13. Vortex the tube for efficient dissolution and transfer the entire 200 μL of 1× assay buffer to the second tube. Again, vortex until pellets from both tubes dissolve completely.

14. Now, the sample is ready for 20E quantification and further use.

*Note: Store the dried 20E pellet at -80 °C. When required, resuspend the pellet to prepare a fresh 20E sample for the assay.*

**Figure 5.**
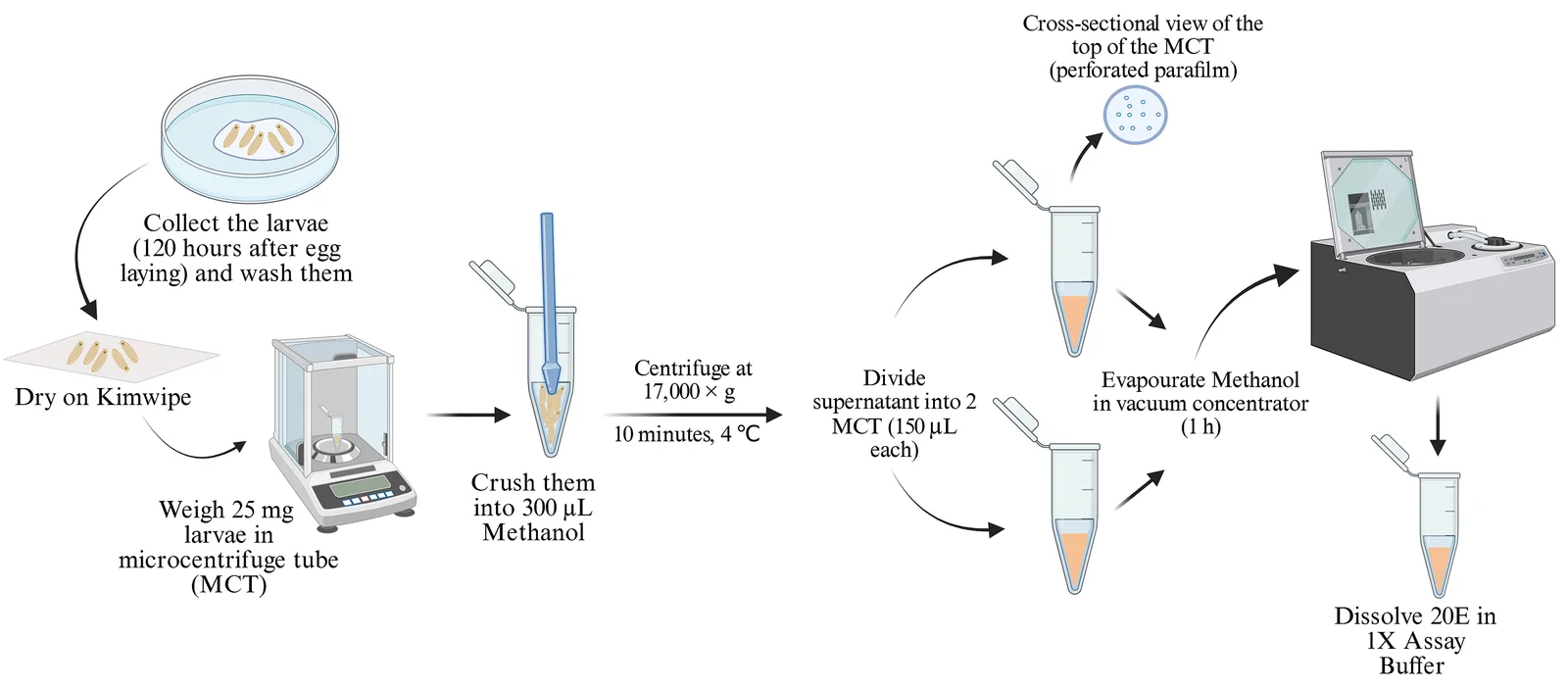
Workflow of sample processing for 20-Hydroxyecdysone (20E) quantification. A stepwise detail of sample preparation from *Drosophila* third-instar larvae.

**E. Measurement of 20E level by enzyme immunoassay (EIA)**

1. To start the ecdysone measurement, keep all the reagents at room temperature and thaw them to equilibrate at room temperature before use.

2. Gently mix the contents of each reagent bottle by inverting it, as provided with the kit. Avoid vortexing.

3. Place the antibody-coated (goat anti-rabbit IgG) 96-well plate, provided in the kit, on a dark surface.

4. In the lab notebook, make an outlay of a 96-well ELISA plate, and label wells in that layout according to Figure S1 to maintain correct reagent addition order.

5. Assign A1 and A2 wells to nonspecific binding (NSB), A3–A12 and B1–B4 wells to different standards (Std1–7), B5–B6 wells to zero standard (B0), and B7–B12 wells to test samples (TS).

*Note: The zero standard (B0) well contains no free 20E. Consequently, all 20E-HRP conjugates bind in this well, resulting in maximum binding.*

6. Add the reagents in a stepwise manner and record each addition in the notebook to ensure accuracy (Table 1).

*Note: Tick off entries in each row/column in your notebook upon adding those reagents to ensure no wells are skipped or cross-contaminated.*

7. At the start, add 75 μL of 1× assay buffer to NSB wells (A1–A2) for detecting nonspecific binding (in duplicate).

8. Dispense 50 μL standards (Std1–7), containing variable amounts of 20E, in duplicate and in triplicate for test samples (TS1–2) wells.

9. To maintain a fixed final volume, add 50 μL of 1× assay buffer to all wells (including NSB) and for detecting maximum binding (zero standard, B0).

10. Add 25 μL of 20E-HRP to each well (including NSB).

11. Lastly, dispense 25 μL of 20E recognizing antibodies to each well except NSB wells.

12. Mix solutions by tapping the side of the plate lightly, and then seal the plate using a plate sealer. Shake at room temperature at 700–900 rpm for 2 h.

*Note: If the plate is not tapped properly, the bound signal intensities could decrease by as much as 20%.*

13. After 2 h, aspirate the solution completely with a pipette from each well and wash wells four times with 300 μL of 1× wash buffer.

14. At the end of the last wash step, empty the plate and pat dry with a paper towel to remove any trace of liquid.

15. Then, add 100 μL of TMB substrate to each well to detect the signal. TMB is a substrate for HRP (horseradish peroxidase), and HRP is conjugated to 20E. HRP activity converts TMB into a product that gives a blue color to the solution.

16. Since the HRP reaction and color generation are light-sensitive, cover the plate with a box and incubate at room temperature for 30 min in the dark.

*Note: Do not use aluminum foil to cover the plate. Hydrochloric acid added during the stop reaction degrades aluminum, which can skew the assay results.*

17. Wipe the bottom of the plate with a paper towel to be sure that no liquid has splashed out of the wells.

18. Add 50 μL of 1 M HCl containing stop solution to each well.

*Note: While handling HCl, always wear appropriate PPE, including a lab coat, safety goggles, and chemical-resistant gloves.*

19. Incubate the plate for 3 min in the dark chamber (cover with a box).

*Note: Maintain dark conditions during this colorimetric assay by covering the plate with a box instead of aluminum foil.*

20. Gently mix the solution either by tapping the plate from all sides or tapping the plate on a flat surface to equilibrate the contents in the well.

*Note: The acidity of HCl turns the blue color into a yellow color.*

21. Record the reading from all wells used in this plate on a BioTek plate reader running BioTek Gen5 software (see Figure 6).

22. Record absorbance values at 450 nm, within 10 min of adding the stop solution.

**Figure 6.**
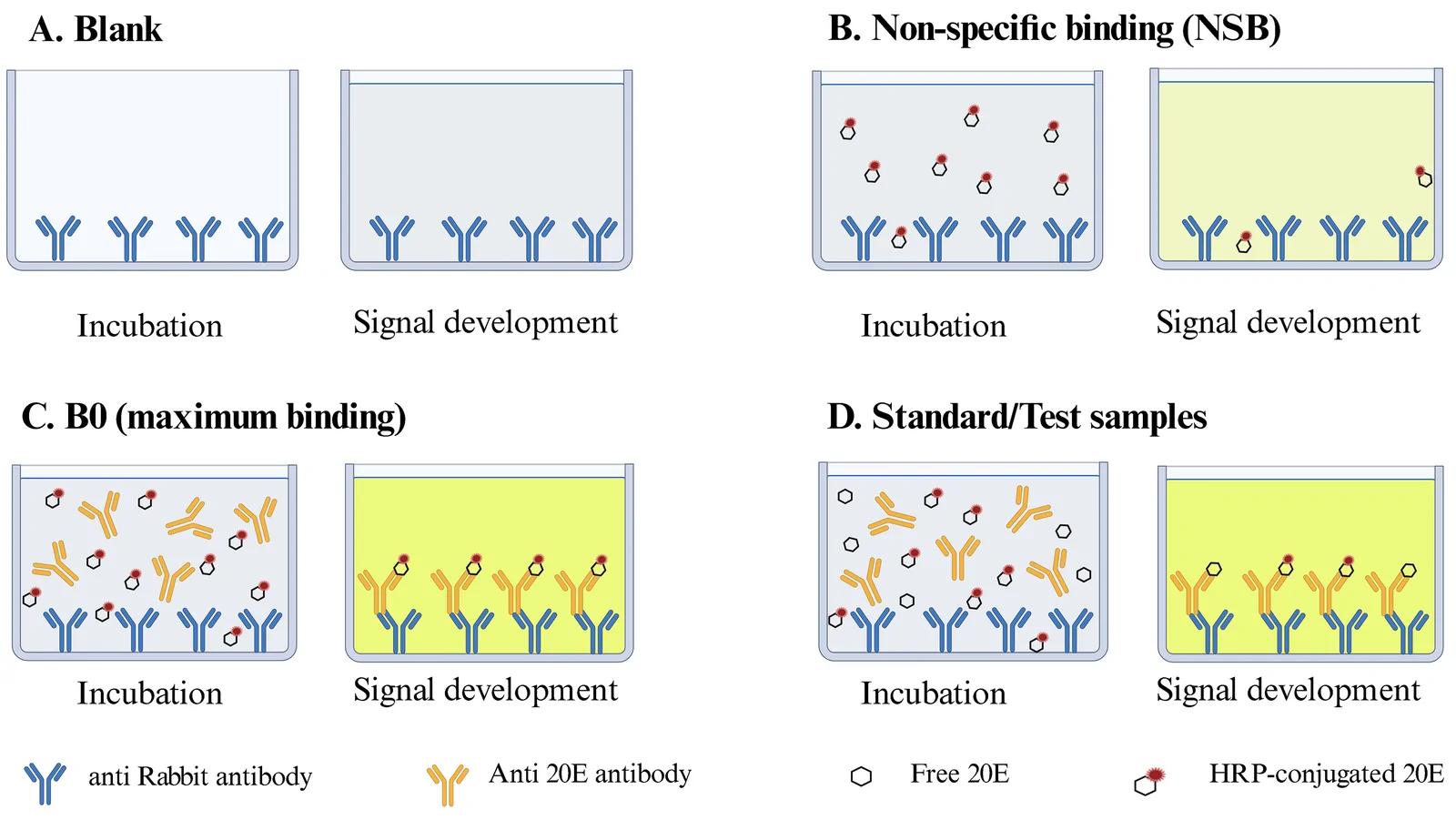
Principle of the ELISA-based 20-Hydroxyecdysone (20E) quantification assay. The principle of 20E estimation by this method relies on competitive ELISA. The presence of unlabeled 20E in samples will compete with 20E-HRP for binding to anti-20E antibodies and enzymatic reaction-dependent color generation. (A) Blank represents anti-rabbit antibody-coated wells without any buffers or reagents (no binding, no reaction, and no color). (B) NSB indicates minimal or nonspecific background binding (nonspecific adherence of 20E-HRP to the substratum and thus background reactivity and color generation). (C) B0 represents the maximum binding of 20E-HRP in the absence of free 20E (no competition, maximum binding, maximum enzymatic activity, highest color generation). (D) Standard demonstrates the inverse relationship between 20E-HRP binding and the concentration of free 20E (available 20E amount-dependent competition, curtailed enzyme activity, reduced color generation).

**Table 1.** Reagent and buffer volumes for competitive ELISA setup

Enzyme immunoassay (volumes in μL)				
	**NSB**	**B0**	**Standard**	**Sample**
**1× assay buffer**	125	50	50	50
**B0**	-	50	-	-
**Standard (Std1–7)**	-	-	50	-
**Sample (TS1–TS2)**	-	-	-	50
**HRP-conjugated 20E**	25	25	25	25
**20E antibody**	-	25	25	25
**Total**	150	150	150	150
Cover plate and shake at room temperature for 2 h at 700–900 rpm				
Wash the plate four times and discard residual liquid from the wells				
**TMB**	100	100	100	100
Cover plate and incubate at room temperature for 30 min				
**Stop solution**	50	50	50	50
Measure absorbance at 450 nm within 10 min of adding the stop solution				

## Data analysis

Obtain the Excel sheet from the plate reader software (generated by the BioTek Gen5 software). Create a new Excel sheet, arrange all the data, and label it properly. Calculate the average absorbance for each well under analysis, such as NSB, B0 (0 pg/mL, without 20E), standards (Std1–7), and samples (TS1–2). Then, subtract the average NSB absorbance from all values. In the absence of any 20E input, B0 will have the maximum binding of 20E-HRP to its antibody, thus the highest absorbance reading. Calculate B/B0 (%) for each standard and sample (average absorbance of standards or sample/average absorbance of B0) and convert to its percentage.

$$\%\frac{B}{B0}=\frac{\text{Standard Absorbance}-\text{NSB}}{\text{Average B0 Absorbance}-\text{NSB}}\times 100$$

Plot a graph with the B/B0 (%) on the y-axis vs. the concentration of 20E (pg/mL) on the x-axis for each standard point. Draw the best-fit line (often logarithmic) through the points. For the calculation of 20E concentration in the test samples, the relevant B/B0 (%) value must be in the range of 20%–80%. Extrapolate these values on the graph to find the value of B/B0 (%) on the y-axis (Figure 7). Read the corresponding value on the x-axis, which is the concentration of the test samples. Alternatively, calculate the x-value (20E concentration) by applying the y-value (absorbance of sample) in the formula y = -14.54ln(x) + 153.83 obtained from plotting the standard values (Figure 7).

**Figure 7.**
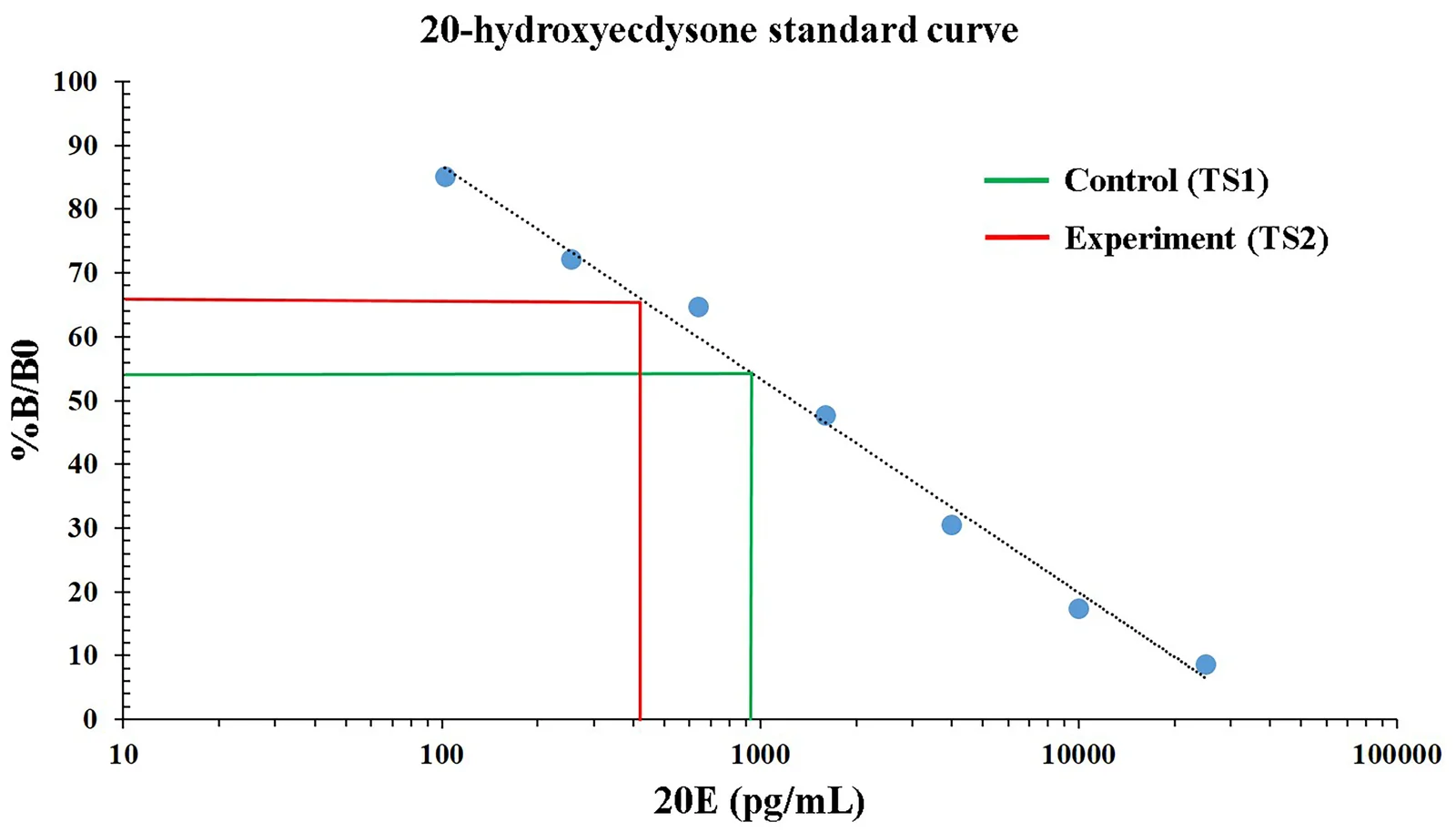
Standard curve of 20-hydroxyecdysone (20E). The standard fitted curve (black dotted line) was obtained by plotting the concentration of 20E (pg/mL) on the x-axis and the % B/B0 (% absorbance) on the y-axis. It also illustrates the comparative analysis of 20E levels between different sample groups. The green line represents the control sample (TS1), while the red line represents the experimental sample (TS2).

*Note: The %B/B0 value for samples falling outside the optimal 20%–80% range will require either more dilution or concentrated sample preparation.*

## Validation of protocol

Quantification of 20E by ELISA is validated only in 1× assay buffer. For the concentration used in making the standard curve, the detection limit of 20E is 102.4 pg/mL, which is the average of the B0 values. The percentage absorbance value for test sample 1 (TS1, the control cross) is 54.5, corresponding to the 926.23 pg/mL concentration of 20E at the late third-instar larval stage (at the onset of metamorphosis). However, values for the test sample 2 (TS2, the experimental cross) are 66.6, which corresponds to 401.31 pg/mL (low level of 20E). This indicates a ~2.31-fold reduction in 20E concentrations in the experimental cross (Figure 7; Figure S2). Due to a low 20E value in experimental crosses, there is a delay in metamorphosis.

Further, this protocol has been used and validated in the following research article: Kawadkar et al. 2026. Nup107 is a crucial regulator of torso-mediated metamorphic transition in *Drosophila melanogaster. eLife* [6].

## General notes and troubleshooting

**General notes**

1. Avoid aluminum foil for covering. This may hamper the smooth progression of the reaction.

2. Use a precisely calibrated pipette for setting up the reactions.

3. Use different micropipette tips for each reagent, and do not expose them to reagents already in the well when setting up the reaction.

4. Completely evaporate all methanol before resuspension in 1× assay buffer; residual methanol will suppress the assay signal and cause erratic results.

5. This assay kit is highly specific for 20-hydroxyecdysone, demonstrating only 0.71% cross-reactivity with precursor ecdysone. Further, only 5% cross-reactivity with 20E analog Makisterone A makes this kit ideal for selective 20E quantification.

**Troubleshooting**

**Problem 1:** Low absorbance values.

Possible causes: Organic contamination from the water, incorrect incubation settings (temperature or duration), abrupt shaking, insufficient reading time, and improper dispensing of 20E-HRP.

Solutions: Use deionized or distilled water to remove organic contamination. Follow the proper temperature and incubation time duration. Carefully dispense 20E-HRP in each well.

**Problem 2:** Background signal in all wells.

Possible cause: Improper washing of the well after incubation steps.

Solution: Increase wash timing, fill the well with wash buffer, and completely decant all residual liquid.

**Problem 3:** High signal in the standard and test wells.

Possible causes: Unusually high signal in one of the turn wells could be due to improper well washing, overdeveloped signal from a long incubation time after adding the stop solution (incubation time should be reduced), or high ambient temperature.

A high signal in the test sample could be due to a high concentration of 20E.

Solution: Follow the recommended time for reading, then wash the well properly. Dilute the test samples a few-fold and reanalyze.

**Problem 4:** High variation in duplicate/triplicate values.

Possible causes: Poor pipetting technique or irregular plate washing.

Solution: Ensure pipetting is performed using a calibrated, high-precision pipette, and that washes are careful and proper.

**Problem 5:** %B/B0 values fall outside the optimal 20%–80% range.

Possible causes: Low/high level of 20E.

Solutions: If the value of %B/B0 is lower than 20%, that suggests a high concentration of 20E in the sample, so the sample should be diluted accordingly and reanalyzed. If the value of %B/B0 is higher than 80%, that suggests a low concentration of 20E in the sample, so the sample should be concentrated accordingly and reanalyzed.

**Problem 6:** Poor standard curve fitting.

Possible cause: Inaccurate serial dilution and insufficient mixing can lead to an unusually low signal (high %B/B0 or high OD) is observed at lower dilutions (e.g., 256 pg/mL 20E gives a %B/B0 value of ~72.1, which is very close to 64.7, the value for the 640 pg/mL concentration). Such inaccuracy will lead to poor standard curve fitting and faulty quantification.

Solution: Prepare a fresh standard solution, reanalyze it, and construct a new standard curve for subsequent use.

## Supplementary information

The following supporting information can be downloaded here:

1. Supplementary File 1: Symbols and units

2. Figure S1: Plate preparation for 20E quantification

3. Figure S2: Calculation of 20E levels in samples
